# The in-vitro effect of gonadotropins’ type and combination on Granulosa cells gene expressions

**DOI:** 10.1186/s12958-022-01017-x

**Published:** 2022-09-24

**Authors:** Yuval Yung, Adva Aizer, Sarah Tieb, Sharon Avhar Maydan, Ettie Maman, Lilach Marom Haham, Jigal Haas, Raoul Orvieto

**Affiliations:** 1 Department of Obstetrics and Gynecology, Chaim Sheba Medical Center, Ramat Gan, Israel; 2 Sackler Faculty of Medicine, Tel-Aviv University, Tel Aviv-Yafo, Israel; 3 The Tarnesby-Tarnowski Chair for Family Planning and Fertility Regulation, Sackler Faculty of Medicine, Tel-Aviv University, Tel Aviv-Yafo, Israel

**Keywords:** Final follicular maturation, Trigger, Gonadotropin, Granulosa cells, Gene expression, Steroidogenesis, IVF

## Abstract

**Objective:**

Nowadays, different modes and timing of GnRH-agonist combined with hCG trigger, for final follicular maturation, have been described. While LH + FSH are the naturally occurring final follicular maturation trigger, hCG is commonly use during stimulated cycle, and recently the introduction of the Dual/Double trigger combines LH + FSH + hCG. In the present study we aim to investigate the messenger RNA (mRNA) expression of reproduction-related genes in human granulosa cells (GCs) exposed to the aforementioned different types and combinations of gonadotropins.

**Material and methods:**

Mural GCs were obtained from follicular fluid aspirated during IVF protocol. GCs were seeded in culture for 4 days with daily medium exchange followed by administration of either hCG (1 U/ml); FSH (1 U/ml) and LH (8 U/ml); or hCG (1 U/ml) and FSH (1 U/ml) and LH (8 U/ml) for 16 h. mRNA was purified from harvested GCs and gene expression was quantitative by qPCR.

**Main outcome measures:**

The expression of genes related to steroidogenesis (StAR/ CYP19) and oocyte maturation (COX2/Amphiregulin) in cultured GCs.

**Results:**

The Dual/Double trigger (LH + FSH + hCG) showed higher activation of steroidogenesis (StAR/CYP19) and maturation (COX2/Amphiregulin) as compared to the naturally occurring trigger (LH + FSH) and the hCG triggers. Moreover, while the naturally occurring trigger (LH + FSH) activated maturation significantly and more intensely than the hCG trigger, no in between group differences were observed with regards to steroidogenic related genes.

**Conclusions:**

Our findings are in agreement with clinical experience, demonstrating the superiority of the double/dual (LH + FSH + hCG) trigger over the naturally occurring and the hCG triggers.

## Introduction

Sufficient estradiol production by the preovulatory follicle induces the mid cycle LH surge, which is followed by a loss of gap junctions between the oocyte and cumulus cells, cumulus expansion, germinal vesicle breakdown, resumption of meiosis and luteinization of the granulosa cells [1]. Moreover, the consequent increase in progesterone synthesis facilitates the positive feedback action of estradiol to induce the concomitant midcycle FSH peak [1]. This peak FSH has several roles, including the assurance of an adequate complement of LH receptors on the granulosa layer and the synthesis of hyaluronic acid matrix that facilitates the expansion and dispersion of the cumulus cells, allowing the oocyte-cumulus cell mass to become free-floating in the antral fluid [1].

As part of a standard/conventional ovarian stimulation (OS) regimen, final follicular maturation is usually triggered by one bolus of human chorionic gonadotropin (hCG) (5000–10,000 units), that is administered as close as possible to the time of ovulation (i.e. 36 hours before oocyte recovery [2]. Human chorionic gonadotropin, a surrogate to the naturally occurring LH surge, induces luteinization of the granulosa cells, final oocyte maturation and resumption of meiosis.

Ovarian hyperstimulation syndrome (OHSS) is a serious complication of OS, almost always occurring either after hCG administration in susceptible patients or during early pregnancy. Individualization of treatment according to the specific risk factors and the specific response in the current cycle has the potential of reducing the risk and the severity of OHSS in susceptible cases [3]. Moreover, OS which combines GnRH antagonist co-treatment and GnRH-agonist (GnRHa) trigger has become a common tool aiming to eliminate severe early OHSS and to support the concept of an OHSS-free clinic [4, 5]. Moreover, following the observations demonstrating comparable or even better oocyte\embryos quality following GnRHa, compared to hCG trigger [6], and the different effects of LH and hCG on the downstream signaling of the LH receptor [7, 8], GnRHa is now offered concomitant to the standard hCG trigger dose to improve oocyte/embryo yield and quality.

Nowadays, different modes and timing of GnRHa (inducing the release of LH and FSH) combined with hCG trigger, for final follicular maturation, have been described [6, 9]. While LH + FSH are the naturally occurring final follicular maturation trigger, hCG is commonly use during stimulated cycle, and recently the introduction of the Dual/Double triggers which combines LH + FSH + hCG to trigger final follicular maturation are widely used [6]. GnRHa and hCG may be offered concomitantly, 35-37 h prior to oocyte retrieval (Dual trigger) or 40 h and 34 h prior to oocyte retrieval, respectively (Double trigger).

Prompted by the aforementioned observations, we aimed to investigate the messenger RNA (mRNA) expression of genes, related to steroidogenesis [StAR(steroidogenic acute regulatory protein)/ CYP19) and oocyte maturation [cyclooxygenase (COX)-2/Amphiregulin), in human granulosa cells (GCs) exposed to the aforementioned different types and combinations of gonadotropins.

## Material and methods

This study consisted of 78 patients undergoing IVF because of male factor infertility or pre-implantation genetic diagnosis. Subjects with BRCA mutations, fragile X disorder, endometriosis, or polycystic ovary syndrome were excluded. GCs were obtained at the time of oocyte retrieval, after receiving signed informed consent from each patient, for the use of cells that would have otherwise been discarded. The study was approved by our local Institutional Review Board (IRB) committee (approval number: SMC-11-6140).

### Mural granulosa cell collection and culture

The method we used for the isolation of the GCs has been previously described [10]. Briefly, expanded mural granulosa cells (MGCs) that surround metaphase II oocytes were collected from pooled follicular fluid, avoiding blood clots, and re-suspended in phosphate-buffered saline (PBS). After allowing the cells to settle by gravity for a few minutes, the top medium was aspirated and the MGCs were re-suspended again with PBS. This step was repeated two to three times until the medium was clear. To decrease variability between women, cells from different patients were pooled together when possible (depending on the number of retrievals that day). Then, PBS was removed and the cells were re-suspended with 1 ml of basic medium [Dulbecco’s modified Eagle’s medium (DMEM/F12, GIBCO, USA)] supplemented with 10% fetal bovine serum and 1% penicillin/streptomycin (GIBCO, USA).

Each pool of cells was divided into four and plated in 12-well plates (100,000 live cells/well) and cultured in basic medium and maintained in culture at 37 °C in a humidified atmosphere of 5% CO2 and 20% O2. After 4 days of culture, with daily medium exchange, each set of 4 wells were cultured for 16 h with fresh basic medium (control), or with either the addition of hCG (1 U/ml) (hCG group), FSH (1 U/ml) and LH (8 U/ml) (GnRHa group), or the combination of hCG (1 U/ml), FSH (1 U/ml) and LH (8 U/ml) (Dual/Double trigger group).

### RNA extraction and qPCR

Total RNA was extracted from MGCs with a micro RNA Isolation kit (Zymo Research Corp, CA USA) according to the manufacturer’s instructions. mRNA purity and concentration were assessed using nanodrop (NanoDrop 2000C, Thermo Scientific Waltham, MA). Total RNA (25 ng) was used for reverse transcription with a high capacity cDNA RT kit (Applied Biosystems, Grand Island NY) in a 10 μl total volume reaction. One microliter of complementary DNA (cDNA) was used per reaction with qRT-PCR being performed with an IQ power SYBR green supermix (Applied Biosystems, Grand Island, NY) using the following step reactions:1 cycle at 95 °C for 20 s as the denaturation step, 40 cycles each at 95 °C for 3 s and then at 60 °C for 30 s. One peak was observed in the melt curve for all primers used. Samples were tested in duplicate with β-actin acting as a housekeeping gene. Details of the primers used are shown in Table 1.

**Table 1:** Primers list – homo sapiens

Gene Name	Primers sequence	Accession#
AREG	sense, 5′- AGCCGACTATGACTACTCAG	NM_001657
	antisense, 5′-CTTAACTACCTGTTCAACTCTGAC	
CYP19A1	sense, 5′- TGGGTCGCCTATCACCAGTAT	NM_000103
	antisense, 5′- TGGTACCGCATGCTCTCATA	
StAR	Sense, 5`- ATTCAAGCTGTGCGCTGGGAGC	NG_011827.1
	antisense, 5` TGGCCATCACAGCCTGTTGCC	
COX2	Sense, 5`- TTCCTCCTGTGCCTGATGAT	NM_000963.4
	antisense, 5` GGGGATCAGGGATGAACTTT	
ACTB	Sense, 5`- ACTCTTCCAGCCTTCCTTCC	NM_000963.4
	antisense, 5` AGCACTGTGTTGGCGTACAG	

### Statistics

Each experiment was carried out at least three times. Data were expressed as mean ± standard error of the mean (SEM) and evaluated with Student’s t-test with a two-tailed distribution, with two samples equaling variance. For all statistical analysis, SPSS 22 software (IBM, Armonk, NY, USA) was used. *P* values < 0.05 were considered statistically significant.

## Results

**Fig. 1:**
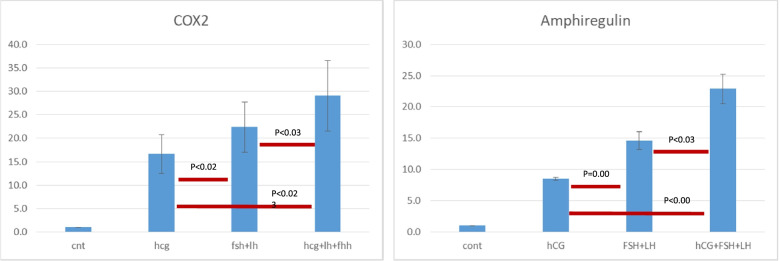
mRNA expression of genes related to maturation

**Fig. 2:**
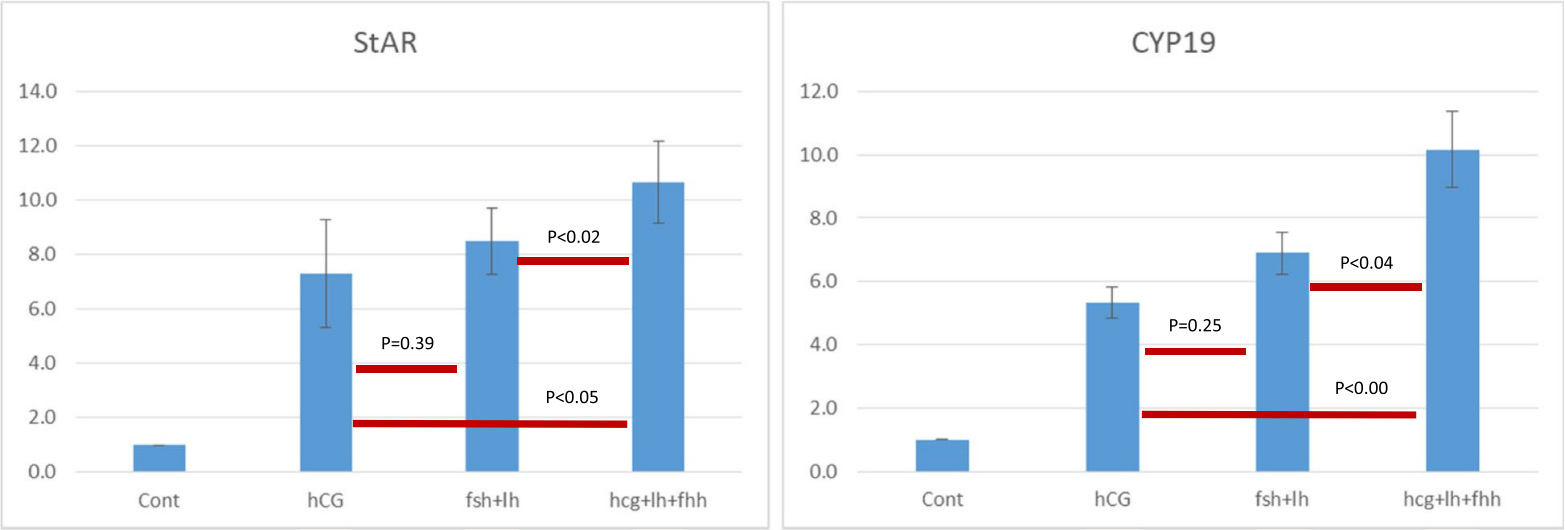
mRNA expression of genes related to steroidogenesis

The Dual/Double trigger group (LH + FSH + hCG) showed significantly higher activation of steroidogenesis (StAR/CYP19) (Fig. 1) and maturation (COX2/Amphiregulin) (Fig. 2) related genes as compared to the naturally occurring trigger (LH + FSH) and the hCG trigger. Moreover, while the naturally occurring trigger (LH + FSH) activated maturation significantly and more intensely than the hCG trigger (Fig. 1), no in between group differences were observed with regards to steroidogenic related genes (Fig. 2).

## Discussion

In the present in-vitro study, the mRNA expression of genes related to steroidogenesis (StAR/ CYP19) and oocyte maturation (COX2/Amphiregulin) were significantly higher following the addition of LH + FSH + hCG compared to LH + FSH or hCG. Moreover, while the expressions of the genes related to maturation were also significantly higher following the addition of LH + FSH compare to the addition of hCG alone, the expressions of those related to steroidogenesis were comparable.

### Oocyte maturation

Amphiregulin is a ligand of the epidermal growth factor receptor, released from MGCs. Luteinizing hormone stimulation of GCs induces up-regulation of amphiregulin, which mediates the LH signal and partially takes part in the process of cumulus expansion and oocyte maturation [11]. Follicle-stimulating hormone has been shown previously to initiate the synthesis of hyaluronic acid, cumulus expansion, and the resumption of meiosis [12–14], and FSH has also been shown to directly increase the mRNA expression of amphiregulin in the GCs [14].

Prostaglandins are essential intrafollicular regulator, required for successful ovulation. Following the mid-cycle LH surge, the level of cyclooxygenase (COX)-2 enzyme increases to reach peak levels near the expected time of ovulation, with the consequent rise in the pre-ovulatory follicular prostaglandin synthesis [15]. Prostaglandins are essential intrafollicular regulator, with a role for successful ovulation.

Our observations confirm previous studies using human GC, demonstrating a higher expression of genes related to follicular maturation (Amphiregulin/COX2) following the exposure to LH [7] or LH + FSH + hCG [8], as compared to hCG. In the present study mRNA expression of amphiregulin and COX2 were significantly higher in the GnRHa and the dual/double trigger groups compared with the hCG group, which might suggest that LH and FSH, or the combination of LH + FSH + hCG induced mRNA expression of amhiregulin and COX2 in different and more intense ways, compared to hCG alone. Ben Ami et al. [16] demonstrated that enrichment of maturation medium with amphiregulin significantly improved the maturation rate of human GV oocytes in vitro. The role of COX-2 in the process of ovulation had previously been reaffirmed by null mouse mutants. COX-2 null mutants are infertile due to severely impaired ovulation. It follows that the increased biosynthesis of ovarian PGs consequent to the LH-triggered induction of ovarian COX-2 activity is essential for the maintenance of a normal ovulatory process [17].

The higher levels of amphiregulin and COX2 following the GnRHa (LH + FSH) or the dual/double trigger (LH + FSH + hCG) may be one of the explanations for the observed advantage of GnRHs compared to hCG. Studies comparing the effects of hCG versus GnRHa triggers on the different follicular maturation variables, following an IVF treatment cycle, have demonstrated that the number of oocytes retrieved, percentage of mature oocytes and number of top-quality embryos were either comparable or in favor of the GnRHa, compared to hCG trigger [6].

### Steroidogenesis

The steroidogenic acute regulatory protein (StAR) predominantly mediates the rate-limiting step in steroid biosynthesis, i.e., the transport of the substrate of all steroid hormones, cholesterol, from the outer to the inner mitochondrial membrane.

Cytochrome P450 aromatase enzyme (P450arom) is the sole member of family 19 of the P450 superfamily of enzymes, termed CYP19 [18]. The enzyme is responsible for estrogen synthesis, by converting androgen precursors to estrogen. Aromatase is the product of the CYP19 gene that is regulated in various tissues by tissue-specific promoters. Aromatase expression in the ovary is regulated by cAMP, producing estradiol, that is the key steroidal hormone regulating feedback control of the gonadotropins.

Our observations demonstrated higher expressions of genes related to steroidogenesis (StAR/CO2) in the dual/double trigger group compared with the LH + FSH and the hCG groups. When comparing the LH + FSH to the hCG groups, no in between group differences were observed with regards to genes related to steroidogenesis. In a previous in-vitro study, Cesarini et al. [7] have demonstrated that LH and hCG stimulate intracellular cAMP accumulation with significantly different kinetics. hCG was shown to exert a more potent effect, as compared to LH on cAMP production, which further stimulates progesterone production. In the present study, while studying the expression of genes related to steroidogenesis (end products), we demonstrated a comparable activation of these genes when adding LH + FSH (the naturally occurring mid-cycle surge) or hCG. It seems that the addition of FSH to LH potentiates the effect of LH on steroidogenesis to the level of hCG. Moreover, the addition of LH + FSH to hCG revealed an additive synergistic benefit over LH + FSH or hCG alone. A previous study by our group [8] has demonstrated no difference in LH and FSH receptor mRNA expressions of MGC retrieved from women undergoing OS for IVF, who were triggered with either hCG or the double trigger. Again, in this study the expression of the LH and FSH receptors were examined, rather than the genes responsible for the end products of steroidogenesis.

## Conclusions

Nowadays, the common real-world practice tailors the mode of final follicular maturation trigger, rather than the OS protocol [6, 9]. Our study further emphasizes and supports the clinical studies demonstrating the advantage of the dual/double trigger in normal [19] and poor responder [20] patients. It actually confirms the following clinical observations. While in patients at risk to develop severe OHSS, GnRHa trigger is offered for final follicular maturation, in those not at risk to develop severe OHSS- three different modes of concomitant administration of both GnRHa and a standard bolus of hCG (5000–10,000 units) prior to oocyte pick-up (OPU) are suggested.

- Dual trigger- both GnRHa and standard hCG administered 35–37 h prior to OPU is offered to normal responder patients, resulting in improved oocyte/embryo quality and IVF outcome [6, 19].
- Double trigger- GnRHa 40 h and standard hCG added 34-36 h prior to OPU is offered to patients demonstrating abnormal final follicular maturation despite normal response to COH, resulting in significantly higher number of oocytes retrieved, higher number of MII oocytes and proportion of MII oocytes per number of oocytes retrieved, with the consequent significantly increased number of top-quality embryos [21, 22].
- Dual trigger 34 h before OPU [9] or double trigger [20] are offered to poor responder patients, aiming to overcome premature luteinization, while achieving high yield of mature oocytes.

## Data Availability

The datasets used and/or analysed during the current study are available from the corresponding author on reasonable request.
